
JOURNAL INFORMATION
==============================
NLM Title Abbreviation: Med Chir Rev
NLM Journal ID: 0034617

The Medico-Chirurgical Review

Publisher: Biomedical Journal Digitization Project

ARTICLE INFORMATION
==============================
PMCID: PMC4966188
PMID: 29917610
Article version: 1
Subjects: Reviews

Dr. Burne on Typhus

Print publication date: 1828 Jul 1
Volume: 9
Issue: 17
First page: 121
Last page: 141
1828 Jun 21
License: This work is free of known copyright restrictions. For more information, please see PMC Back Issue Digitization.
License URL: https://creativecommons.org/publicdomain/mark/1.0/

==============================

### XII.

*A Practical Treatise on the Typhus or Adynamic Fever:* By JOHN BURNE, M. D. &c. &c. Octavo, pp. 248.

PERHAPS there is no class of diseases which has attracted more attention, or given rise to a greater variety of sentiment and discussion than fevers. The frequency of their occurrence, the fatality of their character, and the obscurity in which they are involved, are inducements sufficient to elicit the most patient investigation. But, as in almost every other subject of difficulty, the progress made has not been in proportion to the amount of labour; and, whether we examine their etiology, pathology, or treatment, it is much to be feared that few of our modern writers are more orthodox in their views than the Sage of Cos. The constant bias that exists in our profession to study Nature through the medium of prejudice, to extract general inferences from premises that are particular, and to invest effects with the importance of causes, has cruelly retarded the march of its improvement. It is a science of itself to know how to distinguish facts from fancies, and to ascribe to phenomena, as they rise, the rank and value which Nature has assigned them. Because intestinal disease is a frequent result of fever, it has been inferred by many that fever is its consequence; and, because the brain and its appendages are often found vascular or inflamed, it has been hastily assumed, that the *sedes et causa morbi* are to be found in the brain, and in the brain only. Such exclusive doctrines are the product of partial and undigested views, and the practice they recommend is, consequently, confined to the relief of a few out of many symptoms.

We are, therefore, pleased to find that our present author has taken a more *systematic* view of this disease, and that, while Broussais and Clutterbuck insist upon its *organic* character, we have Burne to add to the number of those, who are not less successful in their practice, while they are more extended in their etiology.

But, while we hail Dr. Burne's escape from the schools of the localists, we fear he has received a lesson out of that of Brown; for, although he refuses to confine fever to a certain seat, he hesitates not to confine it to a certain character. After dividing fevers into such as are inflammatory and adynamic; and subdividing these into such as are purely inflammatory and purely adynamic, and those accompanied by local inflammation, he rejects the generic term *typhus*, to which we have been hitherto accustomed, and adopts the word *adynamic* as a more eligible title, within which he includes "the putrid or malignant fever of Sydenham; the slow nervous fever of Huxham; the nervous fever of common language; the synochus, typhus mitior and gravior, of Cullen; the jail and hospital fever; the fièvres essentielles of the French; the epidemic of the Irish writers; the contagious of Bateman; the typhus of Dr. Armstrong; and the proper, idiopathic, or essential fever of Dr. Clutterbuck." P. 8.

Had Dr. Burne confined his epithet *adynamic* to the lowest and most atonic forms of fever, perhaps, the change, which he wishes to introduce into our nomenclature, would have been useful, and might have been adopted; but we cannot silently subscribe to an alteration, which is founded upon, and inculcates the old doctrine of debility; a doctrine, which, however ornamented by the ingenuity of Brown, and however countenanced by vulgar prejudices, has been productive of infinite mischief to men and to medicine. Holding, as we do, fever, in the abstract, without relation to peculiar epidemics, constitutions, and circumstances, to be more or less a tonic disease, where the whole system is under excitement, and where every texture and organ are stimulated to an inordinate degree, we cannot sanction an epithet which gives the reader, *in limine*, what we deem an erroneous view of its real nature. To designate by the same title the synochus and typhus gravior of Cullen, the putrid fever of Sydenham, and the brain fever of Clutterbuck, is, to say the least of it, indiscriminating and unsafe; not that we imagine they are different in essence, (with Dr. B. we believe them to be the same, "differing only in degree," as he rightly observes, "and modified by circumstances;") but, because the symptoms *are* different, and the treatment *ought* to be different. Our author admits that the debility, which exists in fever, is what Brown would have called *indirect*, and not "ordinary debility," p. 11; but the term *adynamic* (which, in truth, explains itself) signifies not *depression*, but *want* of power, or absolute debility; and, consequently, conveys a meaning very different from that which the writer's own admission required.

We have already stated that Dr. B. following the example of Pinel, divides all fevers into two great classes, viz. inflammatory and adynamic. These he subdivides into simple inflammatory and simple adynamic fever, and inflammatory and adynamic fever complicated with local inflammation: but, as his work is confined to the two varieties of adynamic fever, the observations which follow will, of course, be restricted to them.

"In order to the production (says the Dr.) of the adynamic fever, it is necessary that there be a certain state of system, which state depends, in most instances, on a continued exposure to a poisoned or contaminated atmosphere. This state then existing, it will be found that the adynamic fever attacks in two ways; and, it is of great importance, that these should be clearly understood, because they account for the presence or supervention of inflammation in some cases, and the absence of it in others, and thus afford evidence that certain modern theories are not founded on correct principles. In the one way, then, it attacks through the intervention of an accidental cause; in the other without such intervention. When the attack is without the intervention of an accidental cause, the condition of the body is, of itself, sufficient to stir up and give rise to all those actions and phenomena which constitute the adynamic fever. But, when the attack is with the intervention of an accidental cause, the condition of the body, though not sufficient of itself to produce the fever, is yet sufficient to give to the fever thus accidentally produced, the peculiar adynamic type. In the one case, the development is slow and progressive, requiring many days or even weeks; in the other, it is fully formed in a few hours." P. 14.

He then details the symptoms of attack in both ways, merely differing from each other in the rapidity of their progress, and then remarks, "When it attacks without the intervention of an accidental cause, so far as I have seen, the attack is not accompanied with any organic inflammation;" it being only in such cases as occur through some accidental cause, as a severe cold, exposure to rain, wind, &c. "that organic inflammations are apt to accompany it from the beginning." 17, 18.

The accuracy of these observations we are somewhat disposed to question.* That the febrile poison often lies latent in the constitution for a long time before its existence is announced by appropriate symptoms, is a fact as well established as it is important; and, that the development of these symptoms, in different cases, occupies very different periods of time, is not to be disputed; and, moreover, that there are instances, in which the pathognomonic signs of this disease suddenly and unexpectedly supervene to a perfect state of health, is certain; but, that fever is never accompanied by inflammation, when it attacks without the intervention of an accidental cause, is a position to which we cannot assent. Had Dr. B. required us to believe that inflammation occurred *less frequently* in such cases, his requisition would have been more reasonable; for, it is unquestionable, that the more sudden the excitement is, the less time and power have the weaker organs to resist, or adapt themselves to an increase of action, and the fainter is their chance of escape. We believe there are few examples of what our author calls *simple adynamic fever*, or fever unaccompanied by any organic inflammation. We have inspected many bodies after death, and seldom have we failed in detecting the effects of phlogistic action; and if symptoms during life be minutely watched, we hesitate not to aver, that the experienced observer will generally be able to point out the organ, on which the circulation preys with predominating force. Organic inflammations are not the essentials but accidents of fever, the frequency of whose occurrence is more in proportion to the susceptibility of individual texture, than to the peculiar nature of the exciting cause; and, whether the attacks arise from exposure to cold, or exposure to contagion, the number and nature of the organs affected will mainly depend upon their state of health before the excitement has commenced. Very rarely, we fear, does it happen, that all the parts of our complicated system are equally healthy, and can equally resist the in-

* We imagine that every cause of fever is *accidental*, and that, although some may operate more actively and suddenly than others, fever cannot arise without some *accidental* cause. Respiring a contaminated atmosphere is as much an *accident* as respiring a cold atmosphere; and we are not aware, that any one is exposed, by a *fatal necessity*, to be assailed by this or any other disease. The above division is, therefore, built upon a distinction without a difference.

roads of disease; and we are taught by the simplest law in mechanical philosophy, that the weakest portion of a structure, equally assailed, will be the first to strike to the assailing power.

"The adynamic fever, then, being produced in either of the two ways of attack just described, will be found to differ very much in severity, for which reason and for practical purposes it is expedient to divide it into degrees; and these may, with great propriety, be limited to four." 19. In the first occur slight head-ache, impaired appetite, tongue moist but white, except at the tip, which is red, flushed cheeks, suffused eyes, slight duskiness, and increased heat of skin, red and scanty urine, slow bowels, prostration of strength, and blunted senses. In the second degree the pulse is frequent, "*rather full and rather strong*," the head-ache increases, the tongue is more coated, the skin is hot, (shivering or sense of cold, although one of the most frequent and familiar febrile symptoms, is never mentioned,) the bowels are more inactive, and, in short, the symptoms of the first degree are much aggravated. In the third grade, the strength is much prostrated, the patient is averse to the slightest exertion, the respiration labours, the muscles twitch and tremble, the senses are more torpid, the head-ache becomes more dull, and is often accompanied by a sense of weight or sound, the thirst is excessive, (this is the first time that the symptom is noticed,) the countenance is inexpressive, the flush upon the cheek is now more purple than red, the eyes are suffused, glassy, and vacant, half closed by a relaxation of the upper eye-lids, and besmeared with shreds of mucus, the lips are either blue, or with the teeth covered with black sordes, the breath is very offensive, (there is no notice taken of that peculiar odour which is exhaled from the surface of fever-patients, and than which nothing is more characteristic of this disease,) the tongue is thickly coated, brown and dry in the middle, red and dry at the top, and whitish and moist at the edges, the "*pulse seldom exceeds* 90, *it is fullish, and sometimes rather firm*," the skin is dry and variable in temperature, the urine is turbid when cold, the abdomen is full and tender on heavy pressure, (this is the first notice of a symptom which, we imagine, will be much more frequently found in the two first than the last degrees of fever; for, it is a very important fact, that intestinal disease may go on to a destructive extent, when the sensibility is blunted by an oppressed cerebrum, without betraying even its existence under the very firmest pressure, and this is a circumstance which ought to be largely insisted on, and carefully remembered) the bowels are generally slow, but sometimes lax, and the stools are very offensive; there is much restlessness and delirium, but especially during night. The patient does not, in general, remain long in this state. If he recover, his symptoms either gradually decline, or more rapidly disappear upon the supervention of some critical discharge, as epistaxis, diarrhœa, or a profuse sweat; but, if he sink, his delirium becomes constant, he talks incoherently, or screams, or mutters; his jactitation is incessant, or his prostration so great, that he lies prone and motionless, his lethargy increases, his skin becomes cold and purple, and, the vital function ceasing, the curtain drops. In the fourth and last degree, all the symptoms already specified, become very severe immediately the fever is formed, and run hastily on to a fatal termination; but, as there is nothing peculiar in them, it is unnecessary to follow the author's delineations any further. In a malady so interesting as fever, our investigations cannot be too minute, so long as they are instructive; but, we had rather Dr. B. had traced its symptoms through four different stages than four different degrees, in as far as all these degrees frequently occur during one and the same attack, equally requiring the same variety of treatment.

The second chapter is devoted to some peculiarities from the ordinary combinations of symptoms, for which the author furnishes us with a rationale, by referring to the character of the exciting cause; how, in one case, in which head symptoms had been the most severe, the patient had replaced upon his head a cap that had fallen into the water; and, in another, how the abdomen was principally involved, by the patient having been previously subjected to hard labour. But, why hard labour should not injure the lungs as much as the abdominal viscera, the author leaves us to explain. The truth is, that he might have furnished many such instances of febrile *partialities*, which admit of no elucidation by a reference to the exciting causes, and are only to be accounted for on the principle already stated. The following remarks upon Dr. Armstrong's variety of congestive fever we leave to the consideration of our readers:—

"There is a congestive state also, which, now and then, happens at the beginning, or during the course of the adynamic fever. In this state, there is great and often dangerous depression of the vital powers; so that, instead of hot skin and accelerated pulse, the temperature of the surface is below the natural standard, and the pulse slow and feeble. The skin of the whole body is very dusky, and the hands, feet, and lips of a leaden hue; all, evidently, arising from a congestion of the venous system, the result of feeble and slow circulation; now to this state has been given the term congestive fever, but this is a nosological solecism, for that condition cannot be called fever, in which the signs of fever do not exist; and there is here neither heat of skin nor accelerated pulse, both which are necessary to constitute fever. The appellation is not consistent with the condition specified, and, therefore, is not correct." p. 37.

That the pulse is, in general, the safest index of the activity of the heart, and that the use of the lancet in disease ought generally to be indicated by the energy of this organ, are points in therapeutics which few can question; but, that there may be instances, even in adynamic fever, in which other symptoms than the pulse form more useful, because more practical guides, is an important fact, of which our author seems not to be sufficiently aware. After very considerable depletion, without relief, when the pulse had become *apparently* weak and incompressible, we have seen a few more ounces entirely remove the disagreeable symptoms, and the patient get rapidly well; and, in several instances, we have found, that taking away six or ten ozs. has changed the character of the pulse from that of *masked* debility to strength, requiring further depletion to control it. Our experience, therefore, in such cases, prevents us from adopting any universal rule; and, when we find our author asserting, that in fever "the pulse is never full and strong, and is not firm," (p. 54.) we are still less inclined to receive, without some qualification, his remarks upon a point of so much importance. We are not advocates for bleeding largely in the last stages of fever, nor do we wish to advocate the practice of treating fever, in the first stage, as a *purely* inflammatory disease; but, we maintain that copious and repeated depletions are often safe and even necessary, and, that, while the pulse is sometimes restrained, and simulates weakness through want of bleeding, rising in force and frequency under the use of the lancet, it will frequently be found *full, strong, and firm, not retreating from the finger, nor leaving upon it a slight and transient impression.* Our author will, perhaps, permit us to observe, *en passant*, that his theory of the state of the circulation during fever is built upon several physiological points, which themselves require a foundation. The tonicity of arteries, their power of contracting upon their contents, the assistance given to the venous circulation by muscular contraction, and the necessity of a large column of blood in the arteries to preserve them from collapsing, are positions which have not been proved, and are no more than probable.

In fever, throughout all its degrees and stages, the tongue is an object of much importance, and merits the most particular and constant attention. If carefully watched, it will often indicate, with accuracy, the mucous condition of the stomach and intestines. With this view, the following remarks are very valuable.

"These different states of the tongue correspond with the different states of the alimentary canal. When the tongue is moist, the coating of a light shade and not thick, and the redness of the edges and point not deep, the belly is in a natural state, and so it remains when this tongue becomes clean and of a natural appearance. Where the tongue is dry or parched, the coating dark or black, and the edges and point of a deep red, the belly is flatulent or tympanitic, and tender, in a corresponding degree: the bowels are relaxed, and the dejections dark or black, and highly offensive, constituting the "dark or black offensive diarrhœa" to be spoken of hereafter. When the tongue has cleaned, and is left smooth, moist, of a raw red, and tender, sensible and sore, this state is accompanied with subsidence of the tympanitic belly and with relaxed bowels; but the dejections are now ochre-coloured, and much less offensive, constituting the "ochre-coloured diarrhœa" to be spoken of hereafter. In those instances where, at the decline of the fever, the tongue is left preternaturally clean, but instead of being moist, is dry and shining in the middle and at the point, as if polished, the belly remains tympanitic, more or less; and although the diarrhœa is ochre-coloured, the dejections continue highly offensive, which will be explained when the "ochre-coloured diarrhœa" is considered. When the adynamic fever is combined with rheumatism, the character of the tongue is modified, and the foul surface is made up of a mixture of the dirty coating, peculiar to the adynamic fever, and the white fur peculiar to rheumatism; and, when it becomes clean, the surface will be whiter, and the substance paler, than when rheumatism has not existed." p. 61.

It frequently happens in those cases of fever, which have arisen from, or have been accompanied by neglected bowels, that the abdomen is more or less tympanitic, and betrays considerable tenderness under firm pressure. In such instances, there can be no doubt, that this accumulation of gas is often the product of retained fæces, and will generally disappear with their removal; but, that this tympanitic condition does not *always* arise from constipation is certain, since we daily see febrile patients, after a tardy course of illness, with distended bellies, yet, whose primæ viæ have been carefully cleared out at the commencement of the disease, and preserved regular throughout it by mild aperients. Indeed, the most marked and obstinate cases of tympanitis, which we have witnessed, occurred during convalescence, and, probably, depended upon the debilitated state of the alimentary canal, from a continued course of purging.

Dr. B. in his symptomatology, enumerates diarrhœa, which he distinguishes into two kinds, "the black offensive diarrhœa," and "the ochre-coloured diarrhœa;" the former accompanying the most aggravated periods, and the latter the wane of the disease. But, we much question whether the regularity of this phenomenon entitles it to rank among the common symptoms of fever, and also, whether, when it does appear, its character sanctions such a distinction. Except in the enteritic forms of typhus, where the mucous lining of the bowels is the principal domicile of diseased action, and when its irritabitity is easily excited by the gentlest stimuli, constipation, we apprehend, is a more regular attendant than diarrhœa. If the brain or lungs be chiefly affected, the bowels are generally confined and dull, and seldom, in such cases, do they act without the aid of medicine, unless Nature has chosen them as the outlet, through which, by a critical discharge, she intends expelling the disease. Besides, the external character of the dejections vary daily with the symptoms and progress of the disease; sometimes assuming a more natural, at others a more diseased aspect. During the first stage, while we are unloading the intestines of their accumulated contents, the stools are, in general, both dark and offensive; but, so soon as the discharge has been removed, they put on many varieties of appearance, according to the medicines given, to the inveteracy or mildness of the existing symptoms. Dr. Bright believes that ochre-coloured dejections indicate, either the presence, or approach of ulceration of the bowels; and, although this remark may be disputed as a general observation, we believe experience will warrant the assertion, that, when the intestines are nearly empty, as they are throughout the greater part of fever, treated as it now is with so much opening medicine, and the mucous membrane is excited to an increased secretion, the dejections will be found either yellow, or of a light colour.

The following observations, upon the connexion between ventricular effusion and retention of urine, are, we fear, more ingenious than just.

"There would be little difficulty or attendant danger, was the patient able to direct the attention of the physician to his local distress; but, this he is rendered incapable of, by his general insensibility and confused perceptions. The distention of the bladder, therefore, goes on, and with it, distention of the ureters, infundibula, and pelves of the kidneys. The great pressure of this distention *resists* the distillation of the urine from the mammary processes and uriniferous tubules: the secretion itself is also *thereby* diminished; and *then supervene* the phenomena and effects of suppression of urine; *namely, effusion into the ventricles with its concomitant signs.*

The secretion of urine being only diminished, not altogether suppressed, the bladder would inevitably burst, was it not that the pressure eventually overcomes the resistance of the sphincter, and the urine drips away, forming also incontinence. Yet, notwithstanding this overflowing, the bladder is only secured from bursting, for the great distention and pressure continue, and oppose the secretion of urine, and thereby encourage and augment the ventricular effusion." p. 72.

We believe we may safely assert that the prevailing opinion is the very opposite of that contained in this extract. That deficiency of secretion, or retention of urine may *augment* effusion within the head, it would be unphysiological to deny ; but, that this effusion is the *sole result* of such non-secretion or retention, it is not easy to credit; and it would be more difficult to prove. In every instance, where the urinary apparatus fails to discharge its accustomed duties, the energy and functions of the brain will be found to have been *previously* affected. The mind is either muddled and delirious, or torpid and insensible ; the external senses are either blunted and dull, or cease entirely to acknowledge their appropriate stimuli ; deglutition is impaired, or gone ; the sphincter of the rectum has lost its retentive power ; the patient lies powerless and prostrate, with his head down off the pillow, and his feet drawn up in the bed, and his entire system is under the paralyzing influence of an oppressed, or exhausted sensorium. We do not remember an instance of retention, suppression, or incontinence of urine during fever, in which the cerebral functions were undisturbed ; and we are ignorant of any case, in which either a deficiency, retention, or incontinence of urine *preceded* their disturbance. Derangement of the urinary organ is, therefore, *posterior to, and the effect of* derangement of the brain ; and this rationale of the symptom is confirmed by dissection, when it frequently occurs, that water is found *in large quantities* upon the brain and within the ventricles, while *none lies within the bladder*, and, yet, in such cases no accumulation of urine has been permitted during life, and no difficulty has been experienced in passing it. The reverse of this is likewise no unusual occurrence ; the bladder being found *full of urine, and no effusion within the head*. Moreover, admitting the fact, we would ask our author why the cavities of the brain are those *always* chosen by Nature, into which she may pour the retained fluid ? Why do we not have other forms of dropsy, as hydrothorax, ascites, or anasarca ? In ordinary cases of retained urine, from stricture or diseased prostate, we find no such partiality ; on the contrary, we believe that ventricular effusion is a comparatively rare occurrence.

We have no experience of the state portrayed by the following sentence.

"Sometimes, as the adynamic fever is on the decline, there takes place watching, with a peculiar, staring, rather brilliant, and observant eye, and frequent lifting up of the head, as it were to listen, and mild delirium. The pulse is frequent, generally 120 in the minute ; its stroke is rather open and vibrating, but is short, and leaves no impression on the finger ; and the artery is very compressible. These signs must not be mistaken for irritation, or sub-acute inflammation of the brain : they occur in patients who have lost much blood ; they are the result of that loss, and depend immediately on a defective arterial impulse, and defective supply of blood to the brain." p. 84.

Speaking of the causes which retard convalescence from fever or occasion a relapse, he furnishes us with these important observations:

"The re-establishment of the health of patients, after the adynamic fever has terminated, is, perhaps, more rapid and complete, under favourable circumstances, than after any other disease whatsoever. I have known a convalescent gain flesh after the rate of two pounds in three days, for three weeks successively. Under less favourable circumstance, the period of convalescence is uncertain. At one time it may be early, at another protracted, and is influenced by many causes, such as any unsound condition in which the body may be left, the quantity and quality of the food, the purity or impurity of the air from locality, or the ventilation of the room or ward in which is the convalescent. Whenever an organ has suffered much during the course of an adynamic fever, convalescence is very apt to be protracted, that organ requiring time to resume its healthy functions, or to adapt itself to the new circumstances which may arise out of its diseased condition. Thus, the functions of the brain may remain disturbed for weeks, or those of the lungs be imperfectly performed. Diet, also, influences the period of convalescence; for the weak and susceptible state of the stomach and bowels, together with an eager appetite, render it exceedingly difficult to arrive at health without frequent checks; every little indulgence or excess being immediately followed by general disorder. The continuing to breathe the impure air of a bad locality, or of a close room, or ill ventilated ward (surely these are all localities, and require not specification, after being preceded by a term so general) retards convalescence; and, from this cause, one sometimes sees patients quite at a stand-still, (we know of no variety of standing from that of *standing-still;* standing excludes motion) for many days; convalescents are extremely susceptible of cold, and consequent organic inflammation. I knew a young woman, who lost her life from rheumatic inflammation of the lining membrane of the heart, brought on by exposure to currents of air, and to a damp atmosphere; convalescents are always importunate to be allowed to sit up; but their request must not be readily acceded to." p. 90.

The diagnosis of fever is easy to an experienced observer, but, to those who have witnessed only a few cases, it is occasionally attended with considerable difficulty.

"There are only two affections," says our author, "with which the adynamic fever is likely to be confounded, namely, delirium tremens, and a febrile state which accompanies the latter stages of some diseases of the urinary organs, and there is so much similarity in the disordered condition of the nervous system in all these diseases, that I think it would not be difficult to shew that they are only modifications of the same thing, produced by different causes. They may, nevertheless, be easily distinguished. Delirium tremens is known by the peculiar and excessive tremor of the muscles, from which its name, is, in part, derived; by the very short and breathless respiration, caused by the tremor affecting the diaphragm; and, also, by the vacant, unsteady, and staring roll of the eye; by the constant and extreme agitation, and by the history of the patient's habits. The febrile state, which accompanies affections of the urinary organs, is at once distinguished by the presence of the local disease." p. 102.

There is a circumstance of some importance, however, which is overlooked by Dr. B. in his observations on diagnosis. The physician is not always called in at the commencement of the disease, and often cannot procure such information respecting its incipient symptoms as can be depended on; and, therefore, it is not a rare occurrence that, mistaking the effect for the cause, he pronounces his patient to be labouring under fever, while he is only labouring under the effects of some primary organic inflammation. In the last stages of pneumonia and bronchitis, we have seen symptoms developed, as similar to those of idiopathic fever as it was possible to conceive; and, believing, as we do, that organic inflammation is a much more frequent attendant upon, and consequence of fever than Dr. B. imagines, it is sometimes a task of difficulty to rank cause and effect in their natural relative position, in the absence of a satisfactory history of the case.

In treating of the pathology of fever, Dr. B. divides the morbid changes observable after death, into such as are proper, and such as are accidental, limiting his first class to those of the brain and fecal tube; and then gives directions by which we ought to be guided in conducting our post-mortem researches. They are so correct and important that we will not mutilate them by an extract.

"In all dissections, in which it is desirable to ascertain the exact condition of the vascular system of the brain, the examination should commence with the head; for, if the chest is inspected first, and, as is generally the case, the superior cava or the subclavian veins are divided, the gorged veins and sinuses of the brain will empty themselves through the jugulars into the chest, and so modify very much the appearance. Hence, it is common, under these circumstances, to find the larger veins of the pia mater empty and flaccid, while the smaller are gorged. This emptying of the veins of the pia mater, and of the sinuses, is very much brought about by the great pressure the brain sustains from the force employed to tear off the cranium; a force often sufficient to diminish the conjugate diameter of the brain one inch during the separation of the dura mater. The pressure from this forcible separation acts, in a degree, on the principle of an exhausting pump: it forces the blood out of the veins and sinuses; and when the skull-cap is removed, and the pressure acts no longer, air will not unfrequently find its way through the divided vessels in the chest into the larger veins of the pia mater (?) to supply the place of the blood which has been forced out of them. On the same principle, air will sometimes get into one of the larger veins of the pia mater through a wound in the dura mater, the chest not having been opened." (This is, we think, the true rationale of the appearance of air in the vessels of the head *in all cases*.) "So great an influence has the division of the veins in the chest, in allowing the escape of blood from the venous system of the brain, that I have seen all the blood disgorged from the posterior part of the plexus choroides, leaving its vessel flaccid and empty, while the anterior part remained excessively gorged; the evacuation of the posterior part of the plexus being favoured by gravitation, the body lying on its back, while gravitation opposed the disgorging of the anterior part of the plexus choroides, on account of its peculiar situation and inclination. The morbid appearances depend much on the period at which the dissection is made, and on the cause from which the patient (proximately, we presume, is meant) died; dissection, therefore, should be performed as soon after death as practicable. The bodies of adynamic fever patients seldom grow so stiff after death, as the bodies of those who have died from other diseases. The excessive prostration of the muscular powers peculiar to this fever, prevents the last act of life, the contraction of all the muscles, taking place to the same degree as in other cases." p. 113.

In the head the most ordinary appearances are a sero-gelatinous effusion between the arachnoid and pia mater, opacity and thickening of the arachnoid membrane, (the opacity Dr. B. considers as partly the effect of inflammation, and partly the result of maceration in the fluid lying between it and the pia; but it can only be in such bodies as have been inspected long after death, that we can ascribe much to this latter cause) turgidity of the larger veins and sinuses, except when the serosity is copious,—vascularity of the brain, displayed by making transverse incisions through its substance, when numerous bleeding puncta will appear,—turgescence of the plexus choroides, (this vascular tissue is very seldom highly injected, and never when there is much water within the ventricle) and a variable increase of serum in the ventricles. In some cases the arachnoid is rendered thick by an adventitious deposit, and the fluid beneath it is semi-opake; but he has never seen fibrine effused between the membranes, and although the brain is generally firmer than natural, when the arachnoid is thickened, in common cases it is of the ordinary consistence.

"*When a patient dies from the urgency of the adynamic fever itself, there will be invariably found a greater or less effusion under the arachnoid, with a corresponding one in the ventricles, never exceeding about three drachms.* When, therefore, a much larger quantity is discovered in the ventricles, it may be concluded, that it arises from some other cause than the fever, even though there be effusion under the arachnoid: and, if there is this greater effusion in the ventricles without any effusion under the arachnoid, and an effusion under the arachnoid is invariably found in cases fatal from the urgency of the fever, it follows, *that death*, as also the ventricular effusion, *has been produced by some other cause than the fever, which cause may justly be concluded to be a retention or suppression of urine;* seeing that, in every case of copious ventricular effusion, death has been preceded by one or other of these affections of the urinary organs." p. 121.

The sentiments contained in this passage we dissent from *in toto*, and we caution the inexperienced from adopting them without very mature deliberation. In many cases of the *purest adynamic fever* (and the observations now advanced are the result of multiplied experience) we have found water under the arachnoid *without any effusion* into the ventricles, and, when effusion coexisted with the sub-arachnoid deposit, we have found the quantity to vary from ʒss. to ℥iv. or more; and, that this plus-quantity of ventricular fluid could not have been the product of suppressed or retained urine is rendered indisputable by *the fact*, that, in many such instances, there *was no derangement of the urinary organs.* It is incorrect, therefore, to assert, in terms so unqualified, that, whenever the case proves fatal, through the malignancy of the fever itself, both ventricular and sub-arachnoid effusion will be found, and that the amount of the former will never exceed ʒiij. Besides, admitting that this increased quantity of serum arose from a diminished quantity of urine, will any one be disposed to believe with Dr. B. that death, as well as this ventricular effusion, has been produced by either a retention or suppression of urine? This is certainly "riding a hobby too far." We are not among the number of those, who can find the cause of death *in any case of fever*, an effusion within the head. Such a phenomenon only ranks with us as *one* termination of a preceding action, to which we ascribe much more importance; and we hold it to be a confounding of causes with effect, and our error not confined to our pathology, but extending to our therapeutics, to maintain, that a non-secretion or retention of urine, arising from a palsied condition of the kidneys or bladder, which condition itself arises from a prior derangement of the brain, can, in any case of fever, be the cause of dissolution. Were

Dr. B.'s views correct, very few, indeed, are destroyed immediately by fever. The profession have hitherto nearly overlooked a point involving the most important consequences, and the value of the catheter has not been sufficiently appreciated in its treatment.

Passing over our author's morbid anatomy of the abdomen, which we will refer to in our review of the next article,* we are informed that the bronchial lining is, in general, preternaturally vascular, and has often adhering to it much inspissated mucus, which the patient has not had strength to expectorate. This mucus is sometimes mixed with blood, and sometimes with pus, when the mucous membrane is found thickened and soft, as well as vascular (and may be easily abraded by the nail),—the lungs are, in general, too heavy, and do not completely collapse, their natural structure is not, however, necessarily altered, their inferior and most depending portions are gorged with blood, and are either of a livid or purple colour;—sometimes large portions of them are hepatized and irrespirable, at others, circumscribed livid patches, resembling the spleen in structure, will be discovered in the middle of their substance, and which are considered to have been formed by the effusion of blood before death. The heart is often relaxed and soft, and, in one case, where the symptoms resembled those of delirium tremens, it was found pale, flabby, and easily torn. The inner surface of the aorta will, on some occasions, be seen "of a dark scarlet colour, which may be attributed to staining from the presence of blood in the vessel." The same appearance we have often met in the interior of the heart, especially in its valves and the mouths of the large vessels. p. 139.

Before leaving this department of the subject we wish to observe, that Dr. B. constructs his rationale of the morbid anatomy of fever upon one and the same principle, "debility." If effusion exist within the head it is in consequence of impaired nervous energy; or, if the brain be turgid with blood, it is because its vessels are labouring under a congestion, from deficiency of power to carry forward their contents. If the intestines be found tympanitic, and their mucous tissue inflamed or ulcerated, it is because their muscular powers being prostrated, and their peristaltic action weaker in effect, that their accumulating and putrefying contents extricate thin gases, and irritate the canal to inflammation and disease, or, if the chest betray effects of disorder, whether they be injection or thickening of the bronchial lining, engorgement or consolidation of the lungs, they result, either from inability to expectorate the ordinary secretion, which becomes viscid when retained and irritates the mucous membrane to inflammatory action, or from a dissolved condition of the blood, which, assisted by gravitation and enfeebled respiration, loads the parenchyma of the lungs to a degree incompatible with life.

---

* Review of Dr. Bright's work, which we have been obliged to defer till the next Number.—*Ed.*

This principle (debility) is, therefore, one of great use and applicability with our author; but, while such illustrations of symptoms and disease establish his consistency in adopting the epithet *adynamic*, we question very much, whether their simplicity is a sufficient test of their correctness, or their consistency with the title a satisfactory guarantee of their fidelity to nature. We are strongly inclined to suspect, that effects are sometimes mistaken for causes, and it were as difficult to convince us, that it is viscid mucus which inflames the bronchia, and putrid fæces which ulcerate the intestines, as it were to persuade us that it was the pus expectorated which generated tubercles, or that mucous stools were the cause of diarrhœa.

In another part he observes, that—

"The atonic character of inflammation accompanying the adynamic fever is, moreover, shewn in the products of that inflammation, all of which are nearly destitute of fibrine, the characteristic of tonic or *healthy inflammation*. Where the serous membranes are the seat of inflammation, the effusion, instead of being fibrine, is sero-purulent, with merely shreds or flakes of albumen floating in it; so that there are either no adhesions between these membranes, or the adhesions are partial and slight. Where the mucous membranes are the seat, the product is merely mucus, and seldom or never mucus combined with pus. Where the inflammation is in the parenchyma of any organ, as of the lungs, constituting pneumonia typhodes, the consolidation is in a degree trifling compared with ordinary cases of pneumonic inflammation." p. 42.

Now we can safely assert, that we have seen as active signs of inflammation during life, and as varied results of inflammation after death, in cases of fever, as in any individuals of the order phlegmasiæ. We have seen the pleuræ adhering, nay incorporated as it were; the parenchyma of the lungs fleshy, firm, and irrespirable; the surface of the heart studded with white specks of coagulated lymph; flakes of fibrine floating both in the pleural cavities, and that of the pericardium; the intestines agglutinated so firmly that they were torn by effecting a separation, and several other of the abdominal viscera, as the liver to the diaphragm, the omentum to the spleen, and the fundus of the bladder to the ileum, united by adventitious attachment. Then as to inflammation of the mucous membrane, we have witnessed cases, during which many pints of nearly unmixed pus were expectorated. One we may specify. A woman, of middle age, was seized with the ordinary symptoms of continued fever; but the cavity mainly affected was the chest. She first had a teazing cough, accompanied with mucous sputa; her breathing was hurried, her cheeks gradually became dusky, and then of a leaden hue in the centre; and her dyspnœa increased, and she began to expectorate large quantities of almost pure pus. The purulent expectoration became, at last, so copious, that confirmed consumption was apprehended, and it was only the stethoscope which rectified our diagnosis. She died, and upon dissection, ulceration of the bowels, inflammation, thickening and softening of the mucous membrane of the air tubes, without any change of parenchymatous structure, were discovered.

After controverting in a very ingenious and able manner, the doctrines of Clutterbuck and Broussais, and arguing for the general or systematic nature of fever, the following propositions are laid down :—

"That the adynamic fever has no local seat : that its nature is a morbid condition of the blood, produced by the operation of the primary cause, the respiration of a contaminated or poisoned atmosphere : that the morbid blood, acting on the brain and nervous system, is, of itself, sufficient in very many instances, to bring about the very great derangement and imperfect performance of all the functions of the organic and of the animal life ; which great derangement and imperfect performance of all the functions constitute the phenomena of the adynamic fever." p. 161.

The first proposition we deem incontrovertible by those who have had an extensive experience of fever, and whose minds have neither been warped by theory, nor forestalled by prejudice. The opinions of Broussais, although followed almost exclusively in France, are quite heterodox and untenable ; and, although those of Clutterbuck are more plausible in theory, and more countenanced in practice, they are neither established by symptoms, nor can they explain appearances. But while we concur with our author in rejecting the doctrines of the Localists, as unnatural in not accounting for appearances, as illogical in ranking effects into causes, and as unpractical in fixing the eye of the practitioner upon one symptom and one organ, while it ought to comprehend within its glance every symptom and every organ ; we have not yet determined on adopting his view of the disease, though recommended by its antiquity and the probabilities attending it. In reviving the humoral pathology of fever, Dr. B. ought to have called in the aid of chemistry, as the surest, if not the only means of establishing his doctrine. For, although every symptom and phenomenon receive a satisfactory explanation, by supposing the blood to be diseased, without a confirmation of this supposition, by an analysis of that fluid at the onset and during the progress of fever, such a view could only be regarded as an hypothesis. Had it been proved, that the blood of a fever-patient differs, in its internal constitution and external character, from that of a healthy person,—that this difference is discoverable prior to any other palpable febrile symptom,—that the symptoms proceed in intensity, "pari passu," with this alteration, and that the fever disappears with the disappearance of this difference,—Dr. B. would have had a strong claim upon the faith of his readers, a claim not to be resisted ; but, when no such, and, indeed, no analysis is furnished, when the only observation, made upon the chemistry of febrile blood in the entire volume, disagrees with the results of those who have examined it,* and when we believe, that all the phenomena of fever might be otherwise accounted for, we must for the present, at least, withhold our assent. That the external character of the blood, during the *advanced stages* of this disease, are changed, there is no doubt ; and, that a diseased state of

* See page 142 of this work, and Dr. Clanny's Lectures on Fever.

this fluid might and would produce *symptoms of fever*, we feel no scruple in believing; but, that fever is *in all cases* produced by such an alteration, and that such an alteration exists *before* every other febrile phenomenon, we have several reasons for doubting. At the commencement of the excitement, when the lancet is generally employed, the blood drawn does not differ very materially from that taken out of a healthy system, or one affected by organic inflammation. It separates freely into its constituent parts, and presents frequently, we might say generally, an inflamed surface; but, it is principally as the fever advances, as the whole body becomes more relaxed and disposed to putridity, and as the energies of the nervous system sink, that this fluid assumes a loose, dissolved, and peculiar aspect.

But, having already devoted much space to this review, we must *at present*, abstain from dilating on a subject involved in so much darkness, and which yet requires the most patient and minute investigation, and follow our author to what is equally interesting and not less useful, the treatment of the adynamic fever.

"The treatment of the adynamic fever resolves itself into four principal objects: namely, to arrest the progress of its development; to cure the disease when fully established; to subdue any organic inflammation which may accompany it; to conduct the patient from convalescence to health.

"And, the means by which these objects are to be accomplished, are emetics, aperients, bark, cold-affusion, ventilation, febrifuges, blood-letting, mercury, hyosciamus and opium, stimulants and regimen." p. 166.

"Emetics," he observes, "are *never required*, except when the attack was without the intervention of an accidental cause." p. 166. Now, we really see no good reason for such an exclusive dictum respecting this class of medicines; and, were we friendly to such sweeping generalities, we would have no hesitation in preferring one which conveyed a sentiment the reverse of that contained in this extract. When the fever creeps slowly over the constitution, and gradually takes possession of every organ and function, we believe that neither emetics nor blood-letting will make much, if any impression upon the progress, or character of the disease. In such cases mischief has been imperceptibly accumulating, the functions of life are slowly but surely undermined, indifference gives way to languor, languor to prostration, and prostration to pain. The poison is insinuated, unfelt and by degrees, into the fountain of life; and it is not until its stream be thickly charged, that the unwary patient perceives his preceding disorder was only the commencement of disease. The enemy has gained too firm a grasp of his victim to be surprised and vanquished by a single stroke, and, although an emetic may be occasionally necessary, it will be seldom found to ameliorate the symptoms, or abridge the attack. Not so, however, in cases arising from cold, disordered stomach and bowels, or other accidental causes. The boundaries between health and sickness are here well defined; the moment when the poison began to operate is easily ascertained, and the patient is not surprised into fever by the stealthy mode in which he has been involved. Under such circumstances, if an emetic be *early* employed, it will often arrest the progress of disease, or impart to it a mild and gentle type. We cannot, therefore, sanction the following passage, —when the fever attacks through the intervention of an exciting cause.—

"The development is so sudden, as not to allow the practitioner an opportunity of attempting to arrest it. The development of the fever, therefore, from an accidental cause, cannot be prevented. But, it is far otherwise, when the way of attack is spontaneous, and the development slow and progressive; and if the aid of the practitioner is sought during this period, it will, in very many instances, stifle the fever in its birth, and save the patient from a long and serious illness." p. 200.

A certain time is necessary to habituate the system to a morbid change, and if any thing be done before the formation of this habit, which will impart a sudden and forcible shock, the tendency to disease is frequently destroyed, the chain of morbid action broken, and a new and healthy impulse given to the powers of life. Now, an emetic, or blood-letting, well timed, acts as alteratives, we believe, in some such way; and, having seen many cases prevented and others shortened by such treatment, we consider it necessary to hold forth our veto against a sentiment, in itself, too exclusive, and which, when practically acted on, is calculated to deceive.

Speaking of aperients, he observes, that—

"The best are rhubarb, castor oil, and senna combined with manna. The dose always to be moderate; that of rhubarb varying from five to fifteen grains; of castor oil, from one drachm to half an ounce; of infusion of senna, from one drachm to an ounce, with a proportionate quantity of manna. The larger of these doses are (is) to be prescribed only at the commencement of the disease; and, as a general rule, the longer the disease has existed, the less is the dose required. Although these aperients are all eligible, I have found rhubarb the most so; and so effectually does it answer the purpose, in all states and stages of the disease, that I do not hesitate to recommend its use, to the almost entire exclusion of the castor oil and of the senna." p. 167.

The following passage, we acknowledge, startled us:—

"*Bark is as serviceable in arresting the formation of the adynamic fever, as it is in arresting the progress of an ague; and it may be most advantageously employed when the disease has been for some time on the decline, and distinct remissions occur*; and, also, during the early stages of convalescence, particularly in hospital patients, who have always to contend, more or less, with an impure air." p. 170.

We have already quoted a passage which informs us, that the development of fever from an accidental cause cannot be prevented by any mode of treatment; and, in this extract, we are taught that bark is as effectual in preventing the formation of an adynamic, as it is in curing an intermittent fever; consequently, admitting that one-third of all our cases of continued fever arise from accidental causes, (we use the word *accidental*, of course, in the author's own meaning,) the bark must be supposed to fail in curing one-third of all the cases of ague in which it is tried. Whereas the fact is, that, with some obstinate exceptions, the sulphate of quinine will arrest the progress of ague, as certainly as mercury will arrest the progress of syphilis. This conclusion, however, although drawn from his own premises, we do not imagine he will defend, and, therefore, upon it we will insist no further; but, if bark can cure continued fever as infallibly as intermittent, why is the mortality of typhus so sadly disproportioned to that of ague? There is some obscurity in the structure of this passage, and, therefore, we may be misinterpreting the author. We cannot see how a disease can be *arrested before it is formed;* its *formation* may be *prevented*, and its *progress arrested;* yet, supposing the Dr. intended to convey this meaning, how can he add in the next clause that the bark "may be most advantageously employed when the disease has been for some time on the decline?" Surely, if the formation of the disease can be prevented, or even its progress arrested by bark, it would be most advantageously employed at its commencement, and not at its decline. If we can cut short, or prevent typhus, by the use of this medicine, as certainly as we can cut short or prevent a fit of ague, why allow it to pursue its wonted course? why expose the patient to the pains and hazards of a protracted illness? and why administer at the close, in preference to the outset of the fever, a drug endowed with such therapeutic power? This is, at least, to trifle with sickness and to toy with death.

We attach no such value to bark in the treatment of *continued* fever. It is only when remissions are observable, that it can be employed with any propriety or prospect of success in the *early* stages; if exhibited during the period of excitement, when the skin is hot, the face flushed, the head disordered, the heart labouring, and all the functions disturbed, it will certainly do harm. But, if used in the decline or during convalescence, when the "heat of the battle" is over, and Nature, exhausted by the struggle, only requires strength to "gain the day," we then agree with our author in strongly recommending it, as, perhaps, the most effectual restorative we can have recourse to.

The undeserved neglect which cold-affusion has lately suffered, renders the following judicious remarks peculiarly valuable.

"At any period during the course of the adynamic fever, and in all cases which are not accompanied with local inflammation, cold affusion may be advantageously employed, whenever there is a burning heat of the whole surface of the body; and, particularly, where the skin is very dry, harsh, and contracted, and the prostration of strength great. It diminishes the heat of the surface, saves the strength, disposes the skin to perspiration, and the patient to sleep. I have known delirium cease for several hours after the use of the cold affusion. When the preternatural temperature of the skin is only partial, affusion would be prejudicial; but ablution would be grateful and serviceable; and the hot surface may be sponged with vinegar and water frequently in the course of the day." 173.

We are sorry, after such sentiments, to find that cold lotions to the head are disapproved of.

"The utility of evaporating lotions is, indeed, very questionable, either in these or in more violent cases. I have never seen any decided benefit from them; and they often give cold, and excite a languid inflammation of the eyes, with puriform discharge; and excite or increase pulmonary catarrh." 209.

Perhaps, in one case out of a hundred, the consequences here stated may be produced by such applications, but we have not had experience of them even to that amount; and we are inclined to believe, that, when they do occur, they are not to be attributed to the use, but to the abuse of the remedy. If cold be applied *only* when the temperature of the head is *above* that of the rest of the body, (and it is in such cases only that it will do good,) we are convinced that it will seldom or ever cause any disagreeable result. Evaporation will abstract the excess of heat and not sink the natural temperature, unless too long applied; but, if used when the scalp is not warmer than the rest of the surface, the evaporation will, probably, do more essential mischief by lowering the energy of the brain, than occasion such local inflammation. The consequences, then, are to be ascribed not to the application, but to the practitioner. Exposing the head to a cold atmosphere, after shaving the scalp, will often remove severe head-ache; and increasing the cold by the application of evaporating lotions, or an ice-cap, will frequently remove symptoms the most dangerous and distressing, without the aid of other treatment.

His views of ventilation are equally just and important.

"I have seen, over and over again, patients begin to improve, without the aid of medicine, the moment they have escaped the foul atmosphere of their own dwellings. And this is not to be wondered at, on the belief that the adynamic fever results from a certain condition of the blood, produced by the continued breathing a contaminated or poisoned atmosphere; for then the legitimate inference is, that the unhealthy condition being no longer kept up, but, on the contrary, diminished by the substitution of a pure for an impure air, the effects of that unhealthy condition diminish also, and thus the adynamic signs subside. The impracticability of good ventilation is the reason why it is difficult to cure the poor at their own home; where a whole family, perhaps, is cooped up in a small room filled with dirty, musty furniture, and in which all their little domestic operations are carried on. The adynamic fever, under these circumstances, is always protracted; the efforts of the physician are baffled, and all the remedies which he can administer are barely sufficient to prevent the patient losing ground; much less to conduct him to a safe and speedy convalescence. These are truths which plead in favour of *those noble institutions, hospitals, and declare how necessary they are to the very salvation of the poor afflicted with the adynamic fever.*" 175.

We embrace this opportunity of observing that, although fever is a most prevalent and destructive disease in London, owing to its crowded population, the filthy state of many of its districts, and the wretched habits of its poor; and, although there is *only one hospital* appropriated to fever patients, the support it receives is very limited, and its annual income insufficient to cover expenditure the most reasonable. When we consider the contagious nature of this disease, the ravages which it makes when it enters a family, and the difficulty that is encountered in circumscribing its influence, we cannot help being astonished that an enlightened public can so long overlook the merits of an establishment, to which the lord is equally indebted with the peasant, and which lays claim to a liberal support, as well from the judicious and efficient mode of its management, as from the amount of good which it effects with means so limited.

Had our author confined himself to one form of fever, and had that form been significantly expressed by the term *adynamic*, his remarks upon the treatment in general, and on blood-letting in particular, would have, in our apprehension, been appropriate. But, when we consider that under this epithet he groups every form and degree of this disease, we regard his practice throughout as timorous, and his recommendation of the lancet calculated to discountenance its use. After a few cautious observations in its favour, he winds up the subject by remarking, that "I regret to say, the information, which I have been able to obtain on this point, leads me to the melancholy belief, that, within the last few years, adynamic fever patients have sustained more injury than benefit from the abstraction of blood." 186.

We cannot determine how Dr. B. has arrived at this conclusion, because we have no knowledge of the cases from which it is drawn; but we can safely and certainly aver, that our experience has led us to a very opposite conclusion. We have no doubt but, that during some epidemics, when the type of the disease is characterized by debility, and, in some constitutions, depressed by misery, worn out by nature, or exhausted by fatigue, that the abstraction of blood must either be very limited, or entirely abstained from. But we are not aware that our author makes the above remarks with such peculiarities in view. His language is general and unrestricted, and as such we oppose the doctrine it conveys. Fever, in the abstract, as it has lately appeared and now exists, is not an adynamic affection, although it may become so by the peculiarities of the subject it invades, or of the circumstances attending its attack. Whenever it is seen in the low and *adynamic* type, it is seen modified by some contingent cause, and, therefore, a corresponding modification of treatment will be necessary. But such cases are exceptions to a general rule, variation from the natural portrait; and, although it may be safe and prudent to arrange them into one class, to designate them by a specific title, and to prescribe for them a certain plan of treatment, it is imprudent and unsafe to confuse the exception with the rule, the variation with the standard, and subject a whole genus of disease to curative means only adapted to a species. At the commencement, and during the first stages of fever, blood may, in general, be safely and successfully drawn, apportioning the quantity to the power of the pulse, and the inveteracy of the other symptoms; and we fear that he, who will seal up his lancet-case, and feed his patient upon bark at the outset, will ere long have, at least, as many facts from which to draw a different conclusion, as Dr. B. can advance in support of the one we have extracted.

Our author is opposed to the internal use of mercury, believing it to irritate the mucous membrane of the alimentary canal, and thus dispose to or accelerate ulceration; but, while he prefers rhubarb alone, he admits that there are some instances in which it may be had recourse to with advantage, and strongly recommends its employment externally. As we concur entirely with his views on this point, we shall beg leave to give his sentiments in his own language.

"One of the characteristic conditions of the adynamic fever most difficult to combat, and most desirable to relieve, is the diminution or suspension of the secretions; and the power of the mercurial frictions to restore the secretions exceeds all other remedies, and is, indeed, very remarkable. I have witnessed such speedy convalescence from the operation of mercurial friction, that my mind is quite made up, as to the propriety and utility of this remedy. I believe it may be advantageously employed in all urgent cases of the adynamic fever; and, particularly, when the brain is much affected; as, where there is oppression or lethargy from fulness of blood about the head; where there is restlessness and noisy delirium; or, where inflammatory action is going on; and, indeed, in all cases, and at all times, when the return of the secretion is obstinately protracted. It has quite astonished me, on some occasions, to see how quickly the tongue will cast off its dry brown or black thick coating, the lips and teeth become moist, and the skin soft, and the brain resume its natural functions. Mercurial friction has produced more rapid and favourable changes in very severe cases of the adynamic fever, than any other remedy I have ever seen administered. The average quantity to be rubbed in is half a drachm of the ung. hyd. fort. twice a day." 192.

There is no point connected with the treatment of fever, which is involved in so much obscurity, and requires more judgment and experience, than the use of sedatives and stimuli; and we are not acquainted with one general rule by which to be guided in their administration. We have seen opiates exhibited in cases apparently the most favourable, where constant tremor, jactitation, and watching evinced much nervous irritation, but without any good, and, frequently, with a bad effect; and it has not seldom occurred, that we have witnessed their beneficial operation in instances where the untutored observer would have, à priori, denounced them as misapplied and injurious. The same remark is equally applicable to the use of stimuli in the last stages of fever, and we would recommend the greatest caution in the employment of all such medicines, believing that they invariably do harm, if they fail producing the effect for which they were intended. In cases which seem to require them, they should be at first given in very small doses, increasing, diminishing, or entirely withholding them, according to the nature and amount of the effect produced; as, we are convinced, that the most experienced can seldom tell before-hand whether, by their employment, he is going to relieve his patient, or aggravate his symptoms.

With such views of these medicines we cannot adopt the following rules, that—

"When there is noisy delirium, wakefulness, and restlessness, with an accelerated and easily compressible pulse, hyosciamus or opium may be given, *whatever be the state of the other symptoms*, and will be found valuable remedies. When the adynamic fever has been unusually protracted, and the patient is much exhausted and emaciated, a small allowance of wine, as four ounces, will be serviceable, and accelerate convalescence." 193—196.

In some protracted cases wine may be and is useful, but we object to every thing in the shape of a general rule or principle upon a subject of such obscurity; and, still, of course, excepting peculiar cases and epidemics, we believe it will be found that he who knows most of the nature of fever, and has acquired the *tact* of treating it most successfully, will use the least wine and opium, and, when he does have recourse to them, will use them "with fear and trembling." In the language of our author, "nomine mutato, and if he is in doubt, let the doubt be opposed to the free administration of stimuli." p. 204.

We have now laid before our readers a tolerably minute account of the Doctor's leading views, as to the nature and treatment of fever; and having done so, it is not our intention to follow him through the minutiæ of his practice, as we cannot, by such details, present either much novelty or information. His individual remedies we have individually considered, and the pages which follow he exclusively devotes in applying them to symptoms as they rise, in accordance with the doctrines already reviewed.

We, therefore, dismiss this very unique and ingenious performance with the belief, that the points, in which we differ, arise chiefly from the restricted views our author takes of the nature of fever, and, that could we adopt the doctrine of fever being "in natura et semper" *adynamic*, we could suscribe to many of the sentiments which we have considered it our duty to oppose. But, differing from him, as we do, on this fundamental point, and believing, as we must, from the facts we have seen, and the observations we have made, that fever presents as many varieties of symptoms as there are varieties of causes, constitutions, and circumstances, and that it may appear under every grade of inveteracy, from the most putrid type, where life is seen sinking in a mass of rottenness, up to the most intense phlogistic character, where the lancet must be handled and handled freely ; we have been compelled to impugn many positions that were perfectly consistent with the author's leading doctrine. Fever must not only be seen to be described, but it must be seen under every changing form which changing circumstances can attach to it. In one epidemic its prominent feature its debility, in another it is strength. In one, enteritic symptoms will be found to predominate, in another, disorder of the head. In one constitution every important organ will successively feel the influence of its power, in another, it distributes its excitement in equal proportion throughout the body, passing along its stages without characterizing its attacks or stopping its progress, by any local affection, or leading symptom.

All these varieties must be seen and studied, and every modification of feature must be marked and noted, before we can, either with safety or success, sit down to delineate a faithful portrait. Had Dr. Burne confined himself to a certain epidemic, or to a certain combination of febrile phenomena entitled to the epithet he employs, his work had not occasionally sacrificed its fidelity to Nature for the sake of consistence, and the claims which it certainly has upon public patronage, had not been deteriorated by a comparison of the limited applicability of the principles it inculcates with the extensive magnitude of the subject which it treats.

---

### XIII.

### On Asthma.*

Spasmodic or periodical asthma has been confounded, even by Hoffman, Floyer, and many others, with dyspnœa, or difficulty of breathing, a phenomenon produced by very many organic affections, as chronic catarrh, hepatization of the lungs, hydrothorax, and the various diseases of the heart. It is not difficult to see the mischief which must have resulted from this confounding of affections of the most different kind. But the modern school of Continental pathologists have run into an opposite extreme, and have denied the possibility of a purely spasmodic or periodical asthma—because they have found the same symptoms accompanying organic diseases. The indefatigable Laennec has taken

---

* M. Ferrus—Laennec—Lullier-Winslow—Forbes—Rostan, &c. &c. &c.
